# MPD: a pathogen genome and metagenome database

**DOI:** 10.1093/database/bay055

**Published:** 2018-06-14

**Authors:** Tingting Zhang, Jiaojiao Miao, Na Han, Yujun Qiang, Wen Zhang

**Affiliations:** 1 State Key Laboratory for Infectious Disease Prevention and Control, National Institute for Communicable Disease Control and Prevention, Chinese Center for Disease Control and Prevention, Beijing 102206, China; 2 Collaborative Innovation Center for Diagnosis and Treatment of Infectious Diseases, Hangzhou 310003, China

## Abstract

Advances in high-throughput sequencing have led to unprecedented growth in the amount of available genome sequencing data, especially for bacterial genomes, which has been accompanied by a challenge for the storage and management of such huge datasets. To facilitate bacterial research and related studies, we have developed the Mypathogen database (MPD), which provides access to users for searching, downloading, storing and sharing bacterial genomics data. The MPD represents the first pathogenic database for microbial genomes and metagenomes, and currently covers pathogenic microbial genomes (6604 genera, 11 071 species, 41 906 strains) and metagenomic data from host, air, water and other sources (28 816 samples). The MPD also functions as a management system for statistical and storage data that can be used by different organizations, thereby facilitating data sharing among different organizations and research groups. A user-friendly local client tool is provided to maintain the steady transmission of big sequencing data. The MPD is a useful tool for analysis and management in genomic research, especially for clinical Centers for Disease Control and epidemiological studies, and is expected to contribute to advancing knowledge on pathogenic bacteria genomes and metagenomes.

Database URL: http://data.mypathogen.org

## Introduction

With the rapid development of next-generation sequencing, the enormous amounts of bacterial DNA sequence data continuously emerging have brought forth a challenge for both academic users as well as database curators (1). In particular, the exponential growth rate of biological data has introduced a problem of storage and management for effective research and data sharing. Recently, China has become a powerhouse in generating huge amounts of bacterial genomic data, but is also lacking an effective data management system that can promote the best use of these data and make them publicly accessible to the worldwide scientific community (2, 3).

The increasing amounts of sequence data released in the public domain will be of little value or subject to misinterpretation in comparative analyses without an accurate account of the associated metadata (4). Indeed, availability of high-quality and accurate metadata is an important component for the analysis and interpretation of sequence data. In particular, a database system designed to host a range of pathogenic microbial genomes would be extremely helpful for Centers for Disease Control (CDC), clinical and epidemiological research.

There are currently several available knowledge resources in the field of bacterial genomics and metagenomic, such as the National Center for Biotechnology Information (NCBI) (5), Ensembl (6), European Nucleotide Archive (EMBL-ENA) (7), Human Microbiome Project (HMP) (8), Meta-hit (9), MG-RAST (10) and Imicrobe (11). The NCBI and its international database collaboration partners, the DNA Database of Japan (DDBJ) (12) and EMBL-ENA, strive to collect, manage and distribute these data in the most efficient and usable manner possible. These organizations also provide homology search, database query and information retrieval services for the general molecular biology community as well as more specialized users (13). Unfortunately, the manners in which these data are made available for downloading, homology searching and more general information retrieval purposes are relatively complex and inconsistent among these resources at present. Ensembl and EMBL-ENA focus on different aspects, and thus it is not possible to obtain complete information from a single database. Similar to a metagenomic database, the HMP is focused specifically on the human microbiome, with the goal of investigating how changes in the human microbiome are associated with human health or disease. Meta-Hit specializes on two disorders of increasing importance in Europe, inflammatory bowel disease and obesity, with the aim of establishing associations between the genes of the human intestinal microbiota and health and disease (14). MG-RAST is an open-source web application server that provides support for automatic phylogenetic and functional analysis of metagenome data. Finally, Imicrobe focuses on data obtained from environmental metagenomic studies. Given that the main difference among these databases is the scope of the samples they cover, a comprehensive database is necessary.

To fill this gap, we here introduce the Mypathogen database (MPD), a management system for microbial genomes, which was developed to provide researchers access to searching, downloading and sharing bacterial genomics data, and should be particularly helpful for CDC clinical and epidemiological research. The MPD is a database system designed to host a range of pathogenic microbial genomes and to provide users access to searching, downloading and sharing genomics data. The MPD was created and is maintained by the Bioinformatics Department, State Key Laboratory for Infectious Disease Prevention and Control, Chinese Center for Disease Control and Prevention (China CDC).

## Database implementation

The MPD was developed using J2EE. A browser-based interface was built using D3.js. This database runs on a MySQL relational database, and uses Linux to operate the server system. When designing the website, we coded it in such a way as to reduce dependencies and maximize compatibility with modern desktop browsers (i.e. Firefox, Chrome, 360 and IE).

## Database source

**Figure 1.**
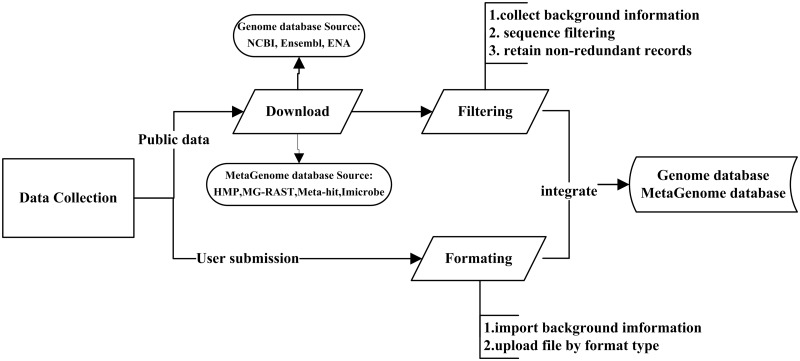
Workflow of the data integration processes. Data are imported from two main sources: public resources and users. For public data: Through the collected background information, some records which lack of background information will be discarded. Then data filtering among different databases was conducted by our self-designed program. Finally, the non-redundant data are uploaded to the integrated genome and metagenome database. For user’s data: Users should input the complete background information, then upload related files with corresponding format types (FASTQ, FASTA and TXT).

The MPD contains publicly available bacterial genomic and metagenomic data, and further serves as an online dynamic visual display tool for microbial genomes. This represents the first database system to host a range of pathogenic microbial genomes (6604 genera, 11 071 species, 41 906 strains) and metagenomic data from host, air, water and other sources (28 816 samples). These public data are being updated on a large scale yearly, and fixing for any data problem at any time. Given that high-quality and accurate metadata are also essential for the analysis and interpretation of sequencing data, this database plays a critical role in filtering manually generated metadata from various resources (public databases or database users) (Figure 1), thereby enabling more useful and meaningful comparative analyses of sequence data.

As shown in Figure 1, data of publicly available genome databases were integrated among the NCBI (15), Ensembl (16) and EMBL-ENA (7), as well as metagenomic databases, including HMP (8), MG-RAST (10), Meta-hit (14) and Imicrobe (11). As the first step, the related information of these genome and metagenome databases (e.g. species relationship, sequencing platform, read length, sample source, reference) is extracted to filter the data. Given the importance of obtaining accurate information of sequencing data for research to effectively compare the public data with newly acquired data, if there is a lack of background information required to carry out the particular research goal, the related file and record will be discarded. Although the bulk of data published in articles are stored in public sequence databases, very often, only raw sequencing data are available; thus, miscellaneous data such as the assembly and genome annotations are not easily obtainable in the same manner. To resolve this issue, the MPD provides convenient methods to download the raw reads, and to also download the related assembled sequence and genome annotation files as well as other files to be analyzed. For example, the genome data downloaded from the NCBI are provided in GBK format, which includes available information on gene annotation. Through the precise information extracted by our self-designed program, the assembled file is generated in FASTA format, along with the associated annotation files in CDS, GFF and PEP format.

The background information of every record is checked for data within each database. If both the project and sample are the same, the redundant files will be removed. Data filtering among different databases was also conducted based on recognizing the names of the projects and samples. When integrating data from the NCBI, Ensembl and EMBL-ENA databases, if identical projects and samples are found, the program is designed to first retain the data from the NCBI, followed by the data from Ensembl. After the data are filtered according to strict rules, the non-redundant data are uploaded to the integrated genome and metagenome database (Workflow of the data filtering is shown in Supplementary Figure S1).

In the MPD database, all genomic and metagenomic raw sequencing data and quality-control data hosted on the website are generated in the standard FASTQ format; the assembled data are in FASTA format; the genome feature annotations are in GFF, CDS, and PEP; and the diversity analysis output for metagenome data are in TXT format. All these data are saved in the international format, which can be easily understood and further used by any user.

The MPD database also supports the saving and management of data submitted directly by academic users, and provides the best protection possible for ensuring the safety of the data. The sequencing data uploaded by users is first retained under a private status, which provides excellent safety and privacy for researchers. The status of the personal data can then be changed according to the owner’s selection (‘Private’, ‘Share’ and ‘Public’). The database supports the following formats for uploaded data files: FASTQ, FASTA and TXT.

**Figure 2.**
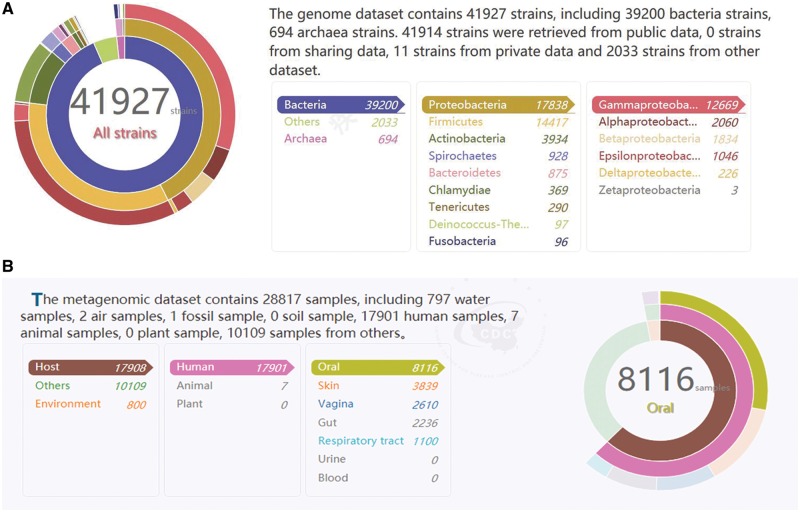
(**A**) The text on the top right is statistics data of the genome database. The three circles from inside to outside is about the number of records according to kingdom, phylum and class in the genome database. It is corresponding to the list on the bottom right. It is a dynamic visual display, when simply drag the mouse on three circles or the list, the other will change with it. (**B**) The text on the top left is statistics data of the metagenome database. The three circles from inside to outside is about the number of records according to sample classification, sample sources and sample sites in the metagenome database. Similar to (A), it is a dynamic visual display and corresponding to the list on the bottom left.

Currently, the MPD database harbors data on a broad range of pathogenic microbial genomes (6604 genera, 11 071 species and 41 926 strains). All of the most popular bacterial species related to human health are represented, including *Escherichia coli*, *Staphylococcus aureus* and *Neisseria meningitidis*. As shown in Figure 2A, the detailed information on the number of records according to kingdom, phylum and class in the genome database is presented in chart form on the homepage. Moreover, the tables and pie charts can be dynamically manipulated for visual representations of the data through a simple click of the mouse to directly display the relevant data list. Each genome is associated with related information such as the sequencing platform used to generate the data, read length, sample source and reference, which is sourced directly from the records in the public databases (NCBI, Ensembl and EMBL-ENA) or submitted directly by other academic users.

In the current version, the MPD also contains 28 816 records on metagenomes representing the microbial communities of different clinical samples (e.g. the human gut, oral cavity, and skin), as well as environmental samples (air, water and soil) (Figure 2B). Similar to the display shown in Figure 2A, the detailed information on the numbers of samples from different sources contained in the database at any given time is displayed in table and pie chart form, which are also dynamic visual representations of the contents. Detailed information about each sample is also provided, such as the sequencing platform used to generate the data, sample source and data files, which can be scanned in the metagenome search page.

## Database use and access

### Search

**Figure 3.**
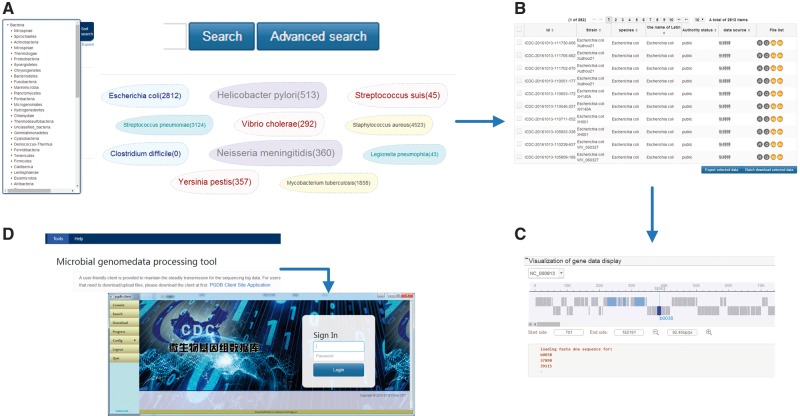
(**A**) Sample search page. The search box on the top is for keyword-based search, which can quick and focus searches; click on the advanced search can allow the user to accurate and customized searches; on the left side is the classification list according to the species relationship and the sample sources in the genome and metagenomic database pages, respectively; in the middle displays the top 10 hot words, which can directly to be linked to the corresponding data. (**B**) Sample search result list. From left to right in the genome result list is the ID number of MPD, strain, species, the name of Latin, authority status, data source and file list. (In the metagenome result list is the ID number of MPD, project information, sample source, sequencing method, authority status, data source and file list.) (**C**) Sample of the online dynamic visual display for microbial genomes. It depends on the genome feature annotation file and the genomic data file, which can scan gene location, length, etc., by simply clicking and dragging the mouse. (**D**) The local client for data transfer. The local client can be downloaded from the tools page on the website, which is provided as a zip file. The user can directly log in to the client by double-clicking the ‘exe’ file contained in the folder after unzipping the file.

Interactive browsing of the sequences available in the MPD is offered through the web interface at http://data.mypathogen.org. The MPD provides four options for users to search the target: (i) keyword-based search, (ii) advanced search, (iii) sidebar-based search and (iv) top ‘hot words’-based search (Figure 3A). For quick and focused searches, we offer a keyword-based search across the genomic and metagenomic databases of the website; users can input partial-matching keywords and receive a list of matches to both databases including relevant information about the file. For accurate and customized searches, we offer the advanced search option, which allows the user to select a variety of search conditions by adding restrictions to more accurately find appropriate data. The classification list for searches is displayed on the left side of both the genome and metagenomic database pages. Moreover, the MPD displays the top 10 hot words in the middle of the page, which are based on the search frequency and research heat, and users can click these words directly to be linked to the corresponding data. Based on search records of users, these hot words will be adjusted and added in the future. Although all sequences can be browsed on the search results page, only ‘Public’ or ‘Share’ records labeled with a term in green are those that can be downloaded. Unless they are meant to be shared by owners, the private data cannot be downloaded by other users of the MPD.

### Genome browser

To best help users understand the genome data more intuitively; the database provides an online dynamic visual display for microbial genomes. The specific display will depend on the genome feature annotation file and the genomic data file. The genome feature annotation is in GFF format, in which the data are provided as plain-text, tab-separated values to ease downstream parsing on the command line and visual inspection via text editors or Microsoft Excel. The genomic data is in FASTA format, which is processed after the assembly. The dynamic visual provides a detailed information page of the strains (Figure 3C), and the user can scan through related information of gene annotation, including the gene location, length, etc., by simply clicking and dragging the mouse.

### Local client for data transfer

A user-friendly client is provided to maintain the steady transmission for the sequencing of big data, as shown in Figure 3D. With this client, users can search, upload and download the corresponding data files using the ID number obtained from a web page search. The client offers maximum safety and stability in the data transmission process. The local client can be downloaded from the tools page on the website, which is provided as a zip file. Moreover, no specific installation is required, and the user can directly log in to the client by double-clicking the ‘exe’ file contained in the folder after unzipping the file.

### Management system

The MPD database also supports a team management function. That is, a department or research team comprising several users with the ability to access and share private data only related to team projects, which will not be available to other users of the database. The data upload status is clearly indicated for users of the same team, and sharing within the team is convenient and safe. To date, the MPD has been used by the China CDC as a genome management system, and has proven to be helpful for sharing data among the different organizations and teams involved.

### The standard operation process (SOP)

The MPD database also provides an integrated information page for SOPs related to next-generation sequencing technology. These SOPs for pathogen detection and identification published by the China CDC currently cover the sample collection, DNA extraction, library construction and sequencing technology. This is based on the experimental process and experience of our own lab, and also integrates certain steps published in related studies. Thus, all of the parameters and steps included in the SOP are subject to strict checks and validation repeatedly. This information will be updated yearly.

## Conclusion

The MPD, a management system for microbial genomes, comprises publicly available bacterial genomic and metagenomic data. It is a pathogen database, and further offers an online dynamic visual display for interpreting and analyzing microbial genomes. The primary mission of the database is to facilitate the analysis and management of datasets for researchers with a log in-free, user-friendly web interface.

The MPD plays a critical role in filtering manually generated metadata from various resources (i.e. public databases or personal users), and these data are constantly being updated. The MPD is expected to develop into a global pathogen bacterial genomic and metagenomic database as more and more data are generated and integrated, and as the related services become increasingly mature.

## Supplementary data

Supplementary data are available at *Database* Online.

## Funding

This study was supported by grants from the Priority Project on Infectious Disease Control and Prevention (2018ZX10303402, 2018ZX10712001, 2018ZX10305410 and 2018ZX10714002), and the National Natural Science Foundation of China (No. 81700016). 

*Conflict of interest*. None declared.

## Supplementary Material

Supplementary Figure
