# Supplementary material for: MPD: a pathogen genome and metagenome database
Source: Database (Oxford). 2018 Jun 14;2018:bay055. doi: 10.1093/database/bay055 (PMC6007212; doi:10.1093/database/bay055)

Supplement Figure 1. Workflow of the data filtering processes. As the first step, the related information of these genome and metagenome databases is extracted to filter the data. If there is a lack of background information, the related file and record will be discarded. Then the related file is needed to generate in standards format by our self-designed program (Using Perl program script to extract the appropriate information and generate files), which the background is complete. The background information of every record is checked for data within each database. If both the project and sample are the same, the redundant files will be removed. Data filtering among different databases was also conducted based on recognizing the names of the projects and samples.


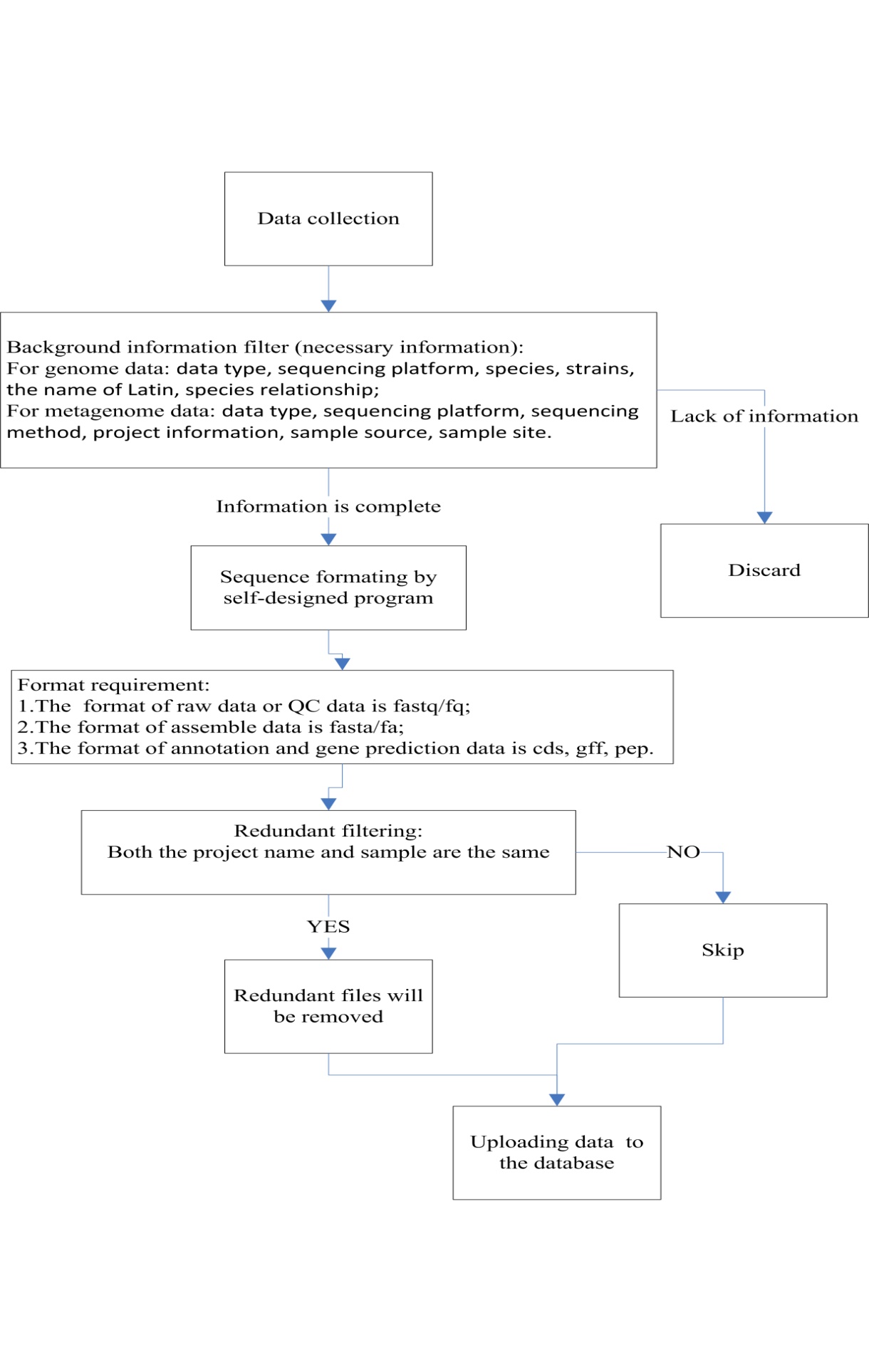

Supplement: Supplementary Figure [file bay055_supp_sf_1.docx]
